# Breast cancer with gastric metastasis in invasive lobular carcinoma: a case report and literature review

**DOI:** 10.3389/fonc.2025.1596207

**Published:** 2025-07-24

**Authors:** Bing Liu, Zhifei Han, Fangqi Hu, Yufei Wang, Xiaofeng Wang, Jiazi Zhang, Jie Chai

**Affiliations:** Department of Gastrointestinal Surgery, Shandong Cancer Hospital and Institute, Shandong First Medical University and Shandong Academy of Medical Sciences, Jinan, Shandong, China

**Keywords:** breast cancer, gastric metastasis, diagnosis, treatment, prognosis

## Abstract

Breast cancer is one of the most common malignant tumors in women. Most early-stage breast cancer does not have the typical symptoms and signs. The common metastatic sites of breast cancer are bones, lymph nodes, soft tissues, lungs, brain, liver. Cases with the gastrointestinal (GI) tract as the first site of metastasis are relatively rare. Their clinical, imaging, and gastroscopic manifestations lack specificity, making them difficult to distinguish from primary gastric cancer. A 45-year-old woman presented with a week-long history of black stools, later diagnosed as gastric metastasis from breast cancer. The patient had undergone surgery, radiotherapy, and chemotherapy 18 years prior for left invasive lobular carcinoma (ILC) of the breast. Current symptoms included black stools, hiccups, fatigue, and decreased appetite, with no nausea, fever, or chills. Gastroscopy revealed a gastric ulcer, and biopsy confirmed poorly differentiated gastric adenocarcinoma. PET-CT indicated high metabolism in the stomach but no distant metastasis. A total gastrectomy with lymph node dissection revealed tumor invasion of the serosal membrane and nerves, confirming metastatic breast cancer. Postoperative treatment and follow-up showed no recurrence or metastasis, and the patient remained stable. Gastric metastasis from breast cancer is an uncommon condition, mostly associated with invasive lobular carcinoma. Accurate diagnosis requires careful consideration of the patient’s medical history and a comprehensive approach utilizing clinical manifestations, imaging, endoscopy, histopathology, and immunohistochemistry (IHC) to minimize the risk of missed or incorrect diagnoses. Treatment remains centered on systemic therapies, including chemotherapy and endocrine therapy.

## Introduction

Breast cancer is the malignant tumor with the highest incidence among women in China (1). It is diagnosed by pathologists It is usually diagnosed by pathologists through histopathological examination after physical examination or screening, and comprehensive treatment is carried out by methods such as surgery, radio, and chemotherapy, targeted and endocrine therapy. The prognosis is affected by multiple factors, including pathological type, disease stage at the time of diagnosis, available molecular markers, and so on. Although early-stage localized breast cancers can achieve the best therapeutic effect after comprehensive treatment, patients’ survival time will be shortened rapidly. Common metastatic sites of breast cancer are local and distant lymph nodes, bones, lungs, liver, and brain (2). Invasive lobular carcinoma has a different metastatic pattern compared to invasive breast carcinoma of no special type. ILC metastasizes more frequently in the gastrointestinal tract, genitourinary system and leptomeninges, whereas pulmonary metastases are less common (3). Based on the available case reports, the GI tract as the first metastatic site of breast cancer is still relatively rare. Research conducted by Xu et al. showed that the incidence rate of gastrointestinal (GI) metastases from breast cancer is relatively low, approximately 0.34% (4). Here, we report the diagnosis and treatment processes of a case of breast cancer with gastric metastasis in Shandong Cancer hospital. We reviewed and analyzed published reports to determine the features of breast cancer pathobiology with GI metastasis.

## Case presentation

**Figure 1:**
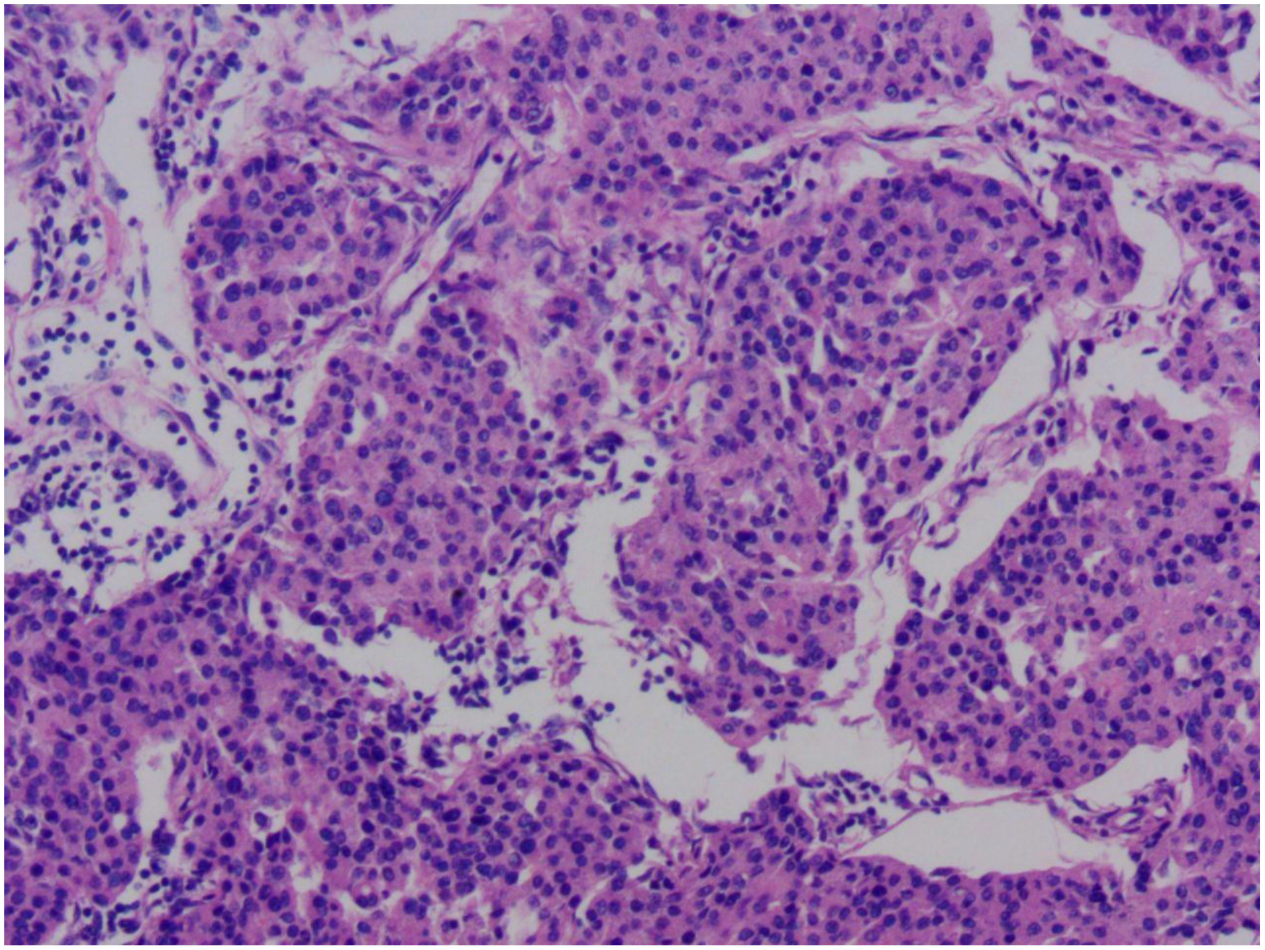
Microscopic pathological image of this patient's breast cancer. The image reveals cancer cells arranged in nest-like structures, with marked nuclear pleomorphism. The size and shape of the cells are inconsistent, demonstrating significant heterogeneity. Normal breast lobular or tubular structures have been disrupted and replaced by disorganized clusters of cancer cells.

**Figure 2:**
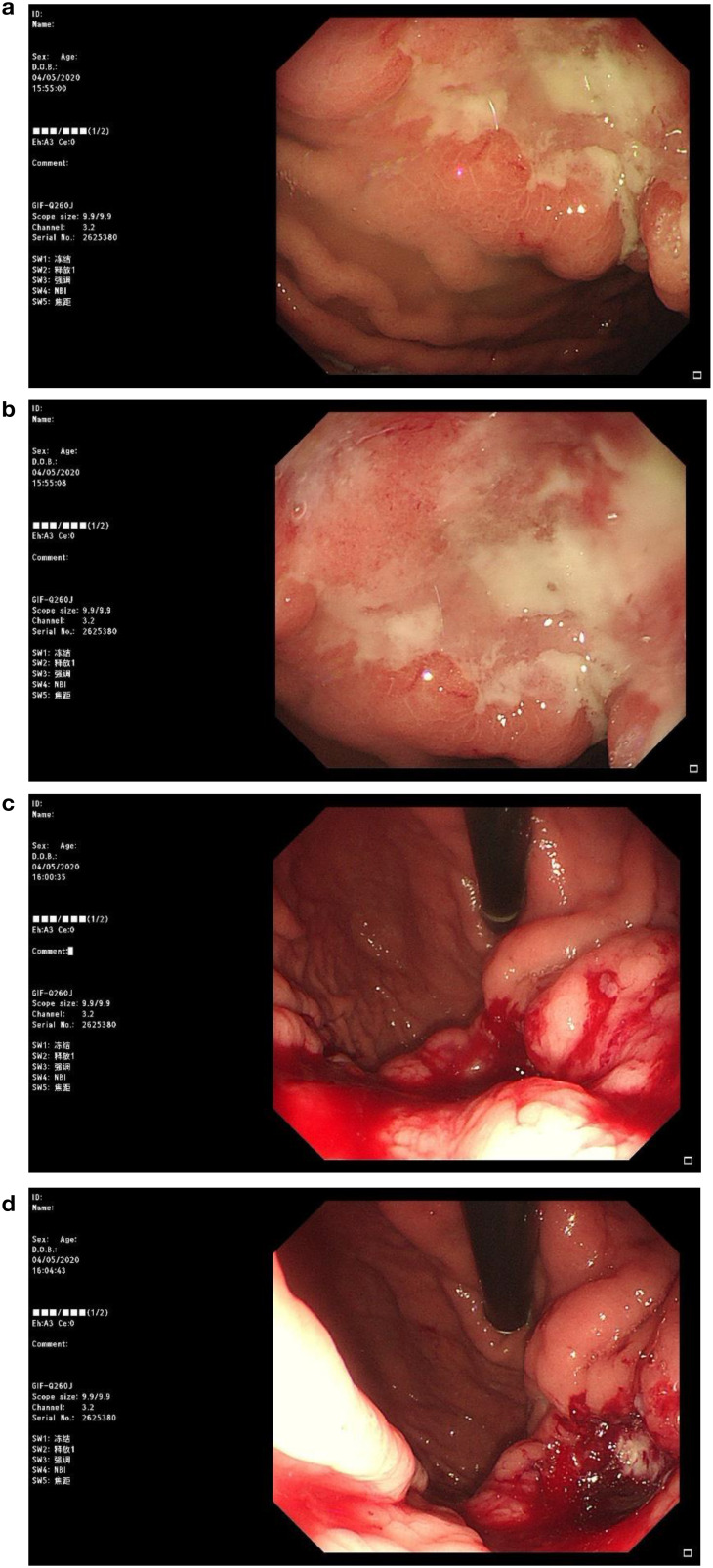
Patient's gastroscopic image. Irregular ulcerative lesions can be observed in figures **(a, b)**, suggesting that the tumor has infiltrated deeper tissues. In figures **(c, d)**, significant active bleeding is evident.

**Figure 3:**
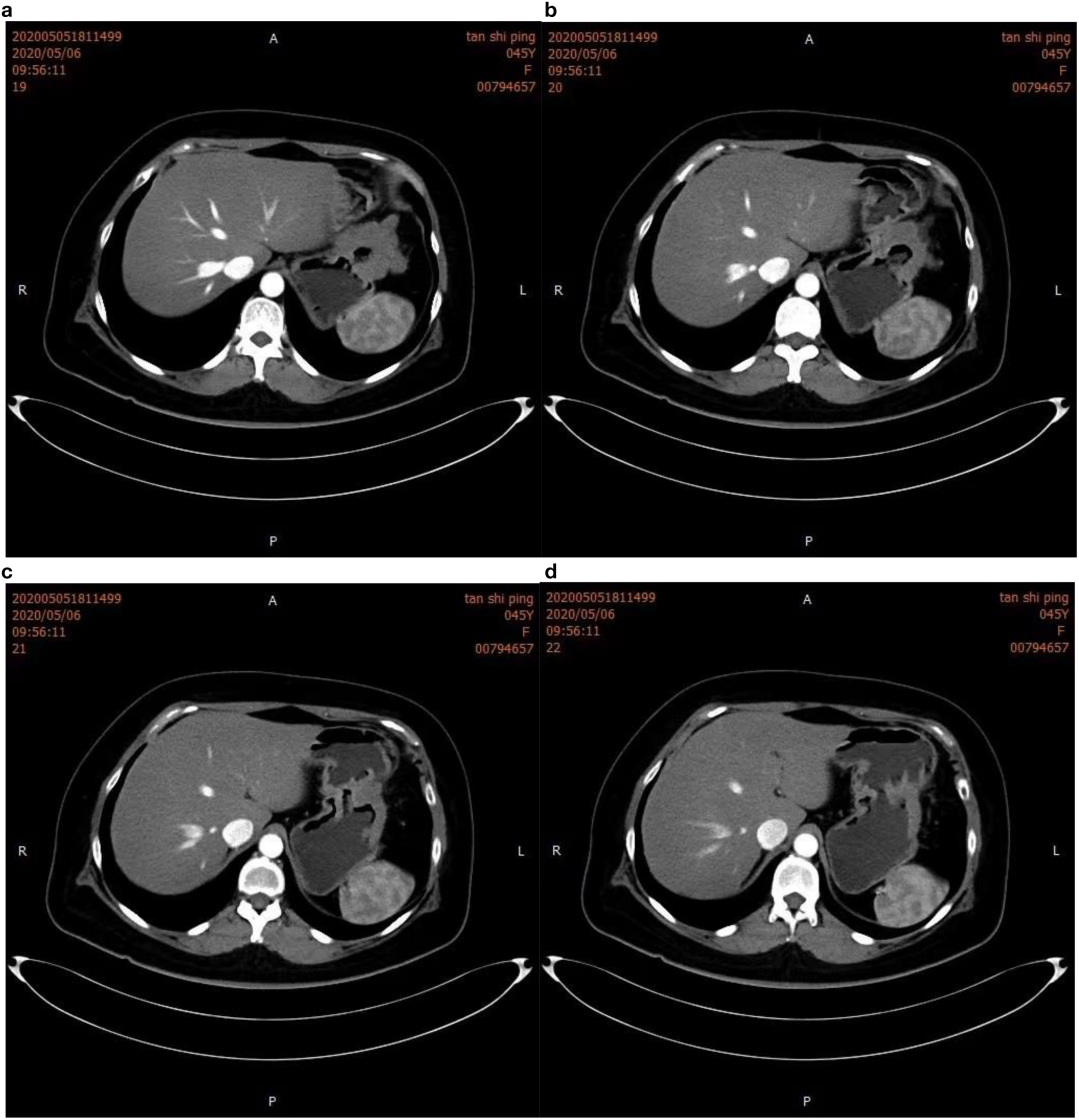
Intensified computed tomography (CT) scan of the abdomen. The image clearly shows gastric wall thickening in figure **(a, b)**, characterized by uneven enhancement. Gastric lumen narrowing is also observed in figure **(c, d)**, suggesting infiltrative growth of the tumor into the gastric cavity.

**Figure 4:**
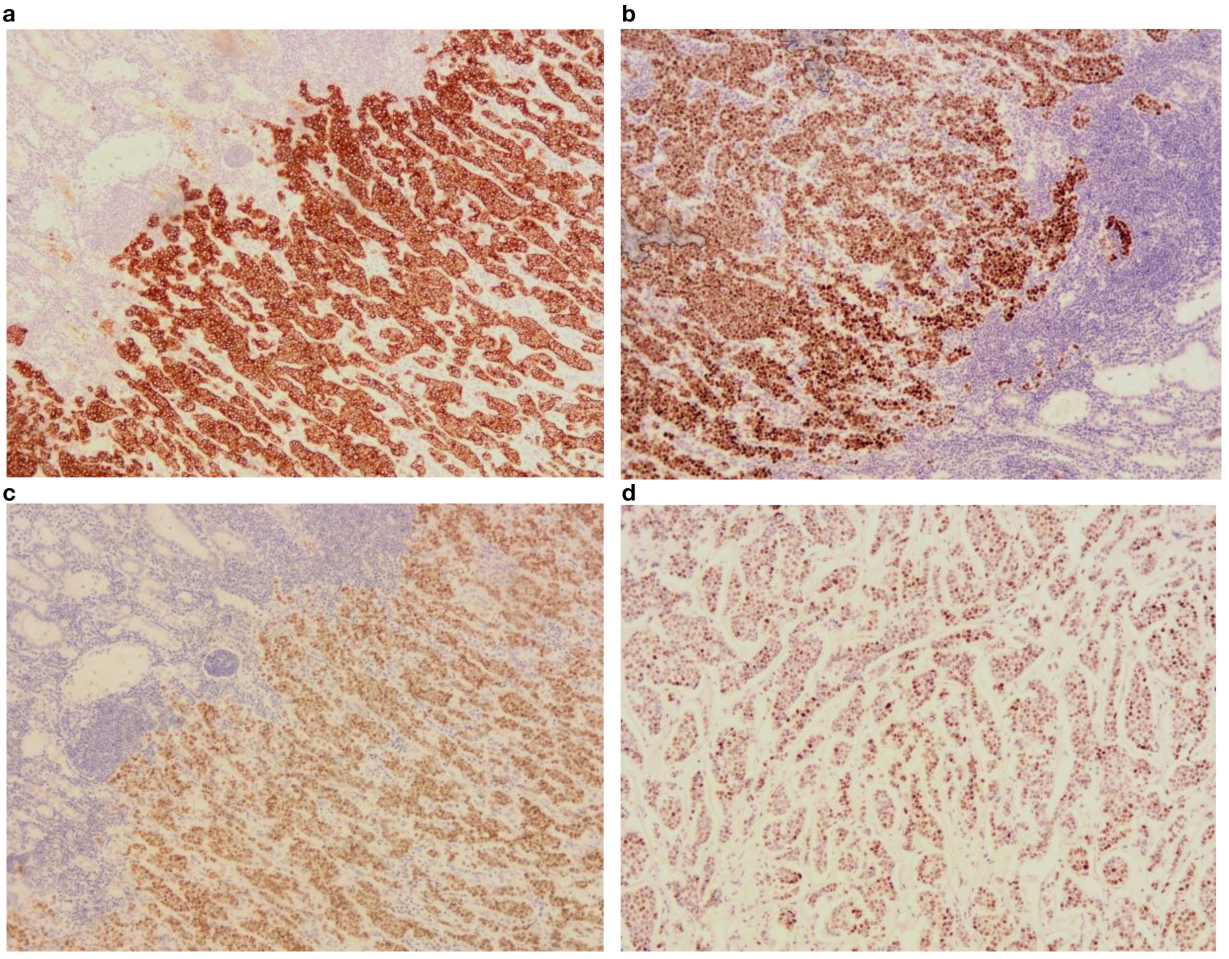
Postoperative immunohistochemical findings under 10x optical microscope. **(a)** CK(+); **(b)** ER(+); **(c)** GATA(+); **(d)** PR(+).

A 45-years-old woman was admitted to our hospital for one-week history of black stool. According to the hospital records, the patient underwent “left breast cancer resection + axillary lymph node dissection” in our hospital 18 years ago (11/19/2002) for “discovering left breast cancer for 3 years”. Postoperative pathology showed the signs of (left) invasive lobular carcinoma of the breast (**Figure 1**). After the surgery, radiotherapy and 6 cycles of regular chemotherapy were given (medication: pirarubicin, the specific dose is unknown), the postoperative recovery was good. In this instance, the patient had no obvious cause of black stools 1 week ago, with an average of 1 time per day, accompanied by hiccups, decreased appetite, as well as fatigue. But no symptoms of nausea and vomiting, fever, and chills, and any other special discomforts were reported. Electronic gastroscopy at the local hospital revealed a huge ulcer in the upper anterior wall of the stomach (**Figure 2**). The pathological results of the hospital showed the signs of poorly differentiated (gastric body) adenocarcinoma. Intensified computed tomography (CT) scan of the abdomen and pelvis at the hospital showed consistent results with the manifestations of gastric cancer in the stomach (**Figure 3**). Pathological results show the poorly differentiated adenocarcinoma (Biopsy of the upper anterior wall of the gastric body). The original unit IHC examinations (**Figure 4**) showed the following results- cytokeratin (CK), CK8/18 (+), CK19 (+), CK7 (+), GATA-binding protein-3 (GATA-3) (+), gross cystic disease fluid protein 15 (GCDFP-15) (+), estrogen receptor (ER) (+) about 70% cells, progesterone receptor (PR) (-), Ki-67 (+) in 10% cells, synaptophysin (Syn) (-), choriogonadotropin alpha chain CGA (-), CD56 (-), and E-cadherin (+). Refer to **Supplementary Table 1** for IHC details. Positron emission tomography-computed tomography (PET-CT) was given to further clarify distant organ metastasis, which showed no abnormal metabolism in the left breast surgery area, while the stomach exhibited high metabolism, indicating a malignant lesion. However, no abnormal metabolism was observed in the abdominal lymph nodes. Laparoscopic-assisted total gastrectomy + abdominal lymph node dissection was performed at Shandong Cancer Hospital on 05/22/2020. Intraoperative exploration showed that the tumor was located in the upper part of the anterior wall of the gastric body, and was ulcer-infiltrated, hard, about 5×4×3 cm in size, penetrated the serosal membrane, and had a clear relationship with the surrounding tissues. There were no obvious swollen lymph nodes around the stomach. Postoperative pathology of total stomach showed the signs of ulcer infiltrating poorly differentiated carcinoma. The tumor invaded the serous membrane and nerve, and tumor thrombus was found in the vessel. The postoperative IHC results further supported the diagnosis of gastric metastasis from breast cancer. Postoperatively, the patient received oral tamoxifen, 1 mg once daily, and tumor control has been good (5). As of the writing of this case report, no recurrence or distant metastasis has been observed during follow-up, and the patient’s overall health condition remained stable. Refer to **Supplementary Figure 1** for a detailed timeline.

## Discussion

Breast cancer is the malignant tumor with the highest incidence among female-associated cancers in the world. According to global cancer statistics 2022, there are about 2,308,897 new cases of female breast cancer all over the world each year (1, 6). The most common pathological type of breast cancer is invasive ductal carcinoma, followed by invasive lobular carcinoma which accounts for around 10% of breast carcinomas (3). Among them, invasive ductal carcinoma is more prone to metastasis to the liver, lung, and brain. The metastatic sites of lobular breast cancer are more inclined to bones, gynecological organs, peritoneum, retroperitoneum, and GI tract (2, 7, 8). Studies have shown that invasive lobular carcinoma leads to gastric metastases in 65.4% of breast cancer cases. The reason for this feature is currently unclear. But overall, the GI tract is relatively rare as the first metastatic site of breast cancer.

Breast cancer metastasized to the stomach often lacks specific clinical manifestations, such as dyspepsia, anorexia, early satiety, upper abdominal pain, vomiting, and hematemesis, which are roughly like the clinical symptoms of GI reactions or primary gastric cancer, thus making it difficult to be distinguished at the initial diagnosis (4, 9). Its common imaging manifestations are localized or diffused thickening of the stomach wall, causing secondary leathery stomach (10). Therefore, it is difficult to distinguish it from primary gastric cancer, gastric stromal tumor, and gastric lymphoma. Tianyue Li et al. (11) have reported that compared to 18F-FDG, 68Ga-FAPI PET/CT showed a significantly higher level of FAPI activity in the thickened gastric wall, and peritoneum. These imaging findings may be helpful for the diagnosis of gastric metastatic cancer (12). Thus, it has a certain reference value, but there are still few imaging studies on indirect invasive gastric metastatic cancer. The gastroscopic changes mainly have three forms, namely 1) volcanic ulcer, 2) single or multiple localized nodules or polyp-like changes in the gastric wall, and 3) limited or diffused gastric wall involvement, gastric wall stiffness, gastric cavity stenosis. Since most gastric metastatic cancers are submucosal with muscular infiltration, endoscopy results may be normal (11). The histopathological findings of gastric metastatic carcinoma also lack specificity. Cancer cells that metastasize to the stomach from lobular breast tumors are often characterized by poorly differentiated adenocarcinoma and signet ring cell-like carcinoma. It is difficult to detect the primary adenocarcinoma of the stomach by the conventional hematoxylin-eosin (H&E) staining of the tumor tissue. In addition, there are reports of gastric cancer metastasis to the breast to interfere with diagnostic judgment. Therefore, the distinction between primary and metastatic gastric cancer must rely on IHC examinations.

In this report, we present a relatively rare case of the invasive lobular carcinoma of the breast that has been metastasized to the stomach. The pathogenesis of this type of metastatic cancer needs to be further explored. Appropriate detection of the expressions of ER, PR, GATA-3, GCDFP-15, mammaglobin, CK7, and CK20 is crucial for the efficient diagnosis of tumor metastasis. GATA-3 is a member of the GATA family of zinc finger protein transcription factors. Because of its important functions in regulating cell development and differentiation, it has been widely used as the breast metastasis marker. Studies have shown its primary expression in breast epithelial tumors (13). In addition, its expression GATA-3 has also been detected in a variety of cancers, including neuroendocrine tumors (14, 15). Although in breast cancer, GATA-3 positive expression ranges from 60% to 100% of tumor cells (13), its expression in metastatic breast cancer is more sensitive and has significance in the differential diagnosis of breast metastasis (14). Despite its potential diagnostic utility, GATA-3 expression specificity is relatively poor in breast metastasis, and which is why it is being utilized in combination with GCDFP-15 and mammaglobin as a biomarker for diagnosis of metastasis. GCDFP-15, a large molecular weight protein, has been found to be positive in 35-74% of cases of breast cancers. The antibody for the detection of GCDFP-15 has strong specificity. CK7 exhibits positive expression in the breast epithelial cells, while negative expression in the intestinal epithelium. However, a small number of gastric cancers can be positive for CK7 expression. E-cadherin is usually expressed in breast ductal carcinoma but is negative in the case of breast lobular carcinoma. Furthermore, new potential diagnostic markers, such as hepatocyte nuclear factor 4a (HNF4a), for the differentiation between primary gastric tumors and metastatic breast carcinoma are also under study (15).

The IHC results of this patient’s tumor tissue exhibited ER (+), PR (+), CK7 (+), GATA-3 (+), E-cadherin (+), and GCDFP-15 (+) tumor cells, which, in combination with patient’s breast examination records strongly proved that the gastric lesions were not the primary ones, rather they were breast metastasized secondary lesions. Furthermore, since the patient provided the medical history of breast cancer upon admission to our hospital, we suggested breast cancer-related antibody testing by IHC, which greatly helped precise diagnosis at the end. Notably, it is difficult to diagnose metastatic breast cancer correctly at the initial stage, especially when the patient’s medical history is unknown. In such cases, digestive endoscopy to examine the appearance of the gastric tumor or lesions can assist in making an appropriate diagnosis. Therefore, the pathological findings from the gastroscopy biopsy should be combined with tumor IHC and other medical examinations to achieve a precise diagnosis of breast metastasis, thereby avoiding undesired mistakes.

The treatment strategies of breast cancer with gastric metastasis are still based on systemic therapy such as chemotherapy and endocrine therapy according to ESMO guidelines (5, 16). Although surgical treatment is the major option for primary gastric cancer, it is not the principal treatment for metastatic gastric cancer. It is often considered palliative treatment when patients have complications, such as GI bleeding, obstruction, and perforation. However, there are also clinical studies that show patients with good general conditions and only gastric metastases demonstrate no statistically significant difference in overall survival prolongation between surgery and chemotherapy (17). Gastric metastasis of breast cancer usually has a poor prognosis, with an average survival time of 41 months (18). Furthermore, the occurrence of gastric metastasis indicates advanced stage with potential spread through the bloodstream to other organs, which means patients’ prognosis in such cases is typically very poor (19). Despite advancements in medical technology improving overall cancer prognosis, GI metastasis remains a challenge in some breast cancer cases. Thus, conducting detailed endoscopy, biopsy, and gathering complete clinical history is essential for diagnosing gastric metastasis cancer accurately and promptly. Simultaneously, accurately distinguishing between primary and secondary gastric tumors is crucial for implementing timely and effective treatment measures to enhance patients’ quality of life.

## Conclusion

Gastric metastasis of breast cancer is a rare occurrence, mostly associated with invasive lobular carcinoma. It is necessary to combine the medical history and comprehensively apply various methods, such as clinical manifestations, imaging, endoscopy, histopathology, and IHC, to prevent missed diagnosis and misdiagnosis. Treatment strategies are still based on systemic treatment such as chemotherapy and endocrine therapy. Palliative surgery may be considered when necessary, but the prognosis remains poor. Therefore, for patients with a history of breast cancer, especially those with invasive lobular carcinoma, who present GI symptoms or are diagnosed with obvious gastric cancer, vigilance regarding possible gastric metastasis from breast tumors is crucial for accurate clinical treatment.

## Data Availability

The original contributions presented in the study are included in the article/**Supplementary Material**. Further inquiries can be directed to the corresponding author/s.
