# Supplementary material for: Breast cancer with gastric metastasis in invasive lobular carcinoma: a case report and literature review
Source: Front Oncol. 2025 Jul 24;15:1596207. doi: 10.3389/fonc.2025.1596207 (PMC12328171; doi:10.3389/fonc.2025.1596207)
Supplement: Supplementary file 1 [file DataSheet1.docx]

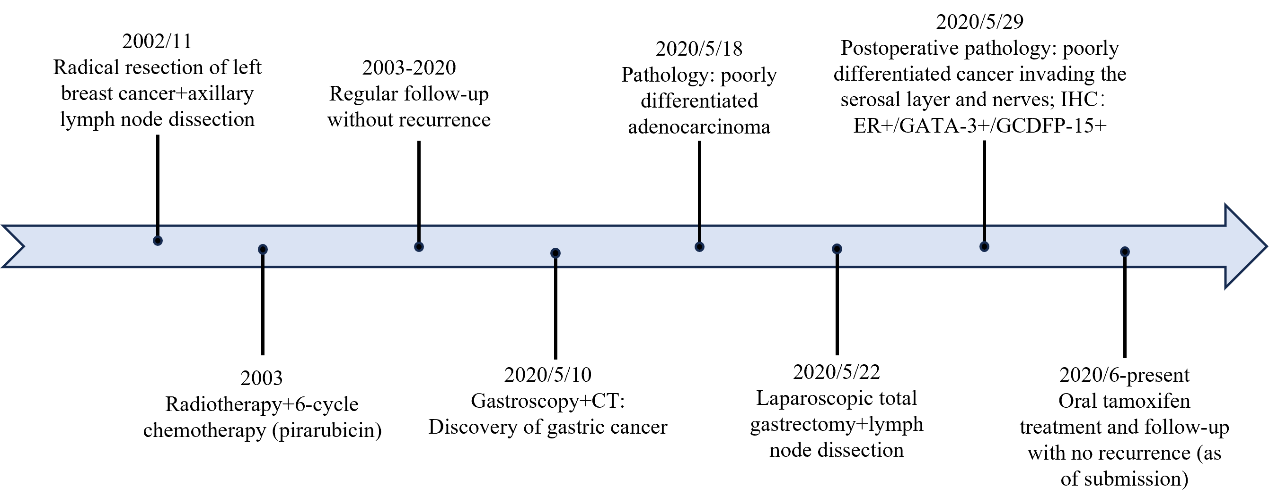


Supplementary_figure_1. The patient's onset and treatment timeline.

| Marker​​ | ​​Result​​ | Interpretation | Diagnostic Utility​ |
| --- | --- | --- | --- |
| ​CK7​ | Positive | Breast epithelial markers | Support breast origin (non-gastrointestinal) |
| ​CK20​ | Not done | (Additional suggestion: should be tested) | Negative results can exclude primary gastric cancer (typical gastric cancer often has CK7-/CK20+) |
| GATA-3 | Positive | Breast Transcription Factor (Sensitivity>95% in ILC) | Breast metastasis specificity>85% |
| GCDFP-15 | Positive | Breast cystic fluid protein (specificity>90%) | Support breast origin |
| ER | Positive | Consistent with primary breast cancer) | Suggests homology with primary tumor clone |
| PR | Negative | Loss (different from primary cancer) | May indicate tumor evolution |
| E-cadherin | Positive | Aberrant expression | Acquired expression in metastatic lesions |
| CDX2 | Not done | (Additional suggestion: should be tested) | Negative can exclude gastrointestinal origin |
| Mammaglobin | Not done | (Additional suggestion: should be tested) | Breast specific marker (complementary to GCDFP-15) |

Supplementary_table_1. Immunohistochemistry Profile and Diagnostic Interpretation​.
